# RNase 7 participates in cutaneous innate control of Corynebacterium amycolatum

**DOI:** 10.1038/s41598-017-14383-z

**Published:** 2017-10-24

**Authors:** Stephanie Walter, Franziska Rademacher, Nicole Kobinger, Maren Simanski, Regine Gläser, Jürgen Harder

**Affiliations:** Department of Dermatology, University of Kiel, Kiel, Germany

## Abstract

Nondiphtheria corynebacteria are typical members of the skin microbiota. However, in addition to being harmless inhabitants of healthy skin commensal skin-derived corynebacteria such as *C. amycolatum* occasionally also cause infections. This suggests that human skin must harbor adequate mechanisms to control the growth of corynebacteria on the skin surface. Here we show that keratinocytes are able to detect the presence of *C. amycolatum* leading to the epidermal growth factor receptor (EGFR)-dependent induction of the antimicrobial protein RNase 7. *C. amycolatum*-mediated induction of RNase 7 was also confirmed in a human 3D skin equivalent. The functional relevance of these findings was demonstrated by potent antimicrobial activity of RNase 7 against *C. amycolatum* and *C. xerosis*. In addition, the capacity of human stratum corneum to restrict the growth of *C. amycolatum* was significantly attenuated when RNase 7 was inactivated by a specific RNase 7-neutralizing antibody. Taken together, the interaction of RNase 7 with *C. amycolatum* indicates that RNase 7 may function as important effector molecule to control the growth of corynebacteria on human skin.

## Introduction

Nondiphtheriae corynebacteria are abundant members of the normal microbiota of human healthy skin and mucosal surfaces. It is known that aerobic corynebacteria contribute to axillary malodor by biotransformation of sweat components^1^. There are various reports demonstrating that nondiphtheria corynebacteria are also able to cause various infections such as skin and soft tissue infections, granulomatous lymphadenitis, pneumonitis, pharyngitis and endocarditis, especially in immunocompromised patients^2–5^.

*Corynebacterium amycolatum* has been originally isolated from human skin as a normal member of the cutaneous microbiota^6^. Although *C. amycolatum* is a normal inhabitant of human skin there is increasing evidence that *C. amycolatum* has the capacity to act as an opportunistic pathogen causing skin and soft tissue infections^2^. *C. amycolatum* has also been associated with wound infections and surgical site infections^7,8^. This suggests that the normal presence of *C. amycolatum* on the skin surface should be tightly controlled.

Antimicrobial peptides (AMP) are known for their potential to control the growth of microorganisms on the skin surface^9–11^. Since the interaction of AMP with corynebacteria has not yet been investigated we sought to determine whether AMP may play a role to control the growth of corynebacteria. RNase 7 is an important skin-derived AMP abundantly expressed by keratinocytes^12,13^. Its broad spectrum of antimicrobial activity together with its abundance in the uppermost epidermal layers indicates that RNase 7 plays an important role in cutaneous innate defense^14,15^. However, there are no reports available exploring the interaction of corynebacteria with human keratinocytes and RNase 7.

Here we show an epidermal growth factor receptor (EGFR)-dependent induction of RNase 7 in primary keratinocytes stimulated with *C. amycolatum*. In addition, *C. amycolatum* induced RNase 7 also in a 3D skin equivalent. We further demonstrate that RNase 7 exhibits antimicrobial activity against corynebacteria and contributes to the capacity of human stratum corneum to control the growth of *C. amycolatum*. These data show for the first time that corynebacteria are able to activate human keratinocytes and identify RNase 7 as an important mediator to control the growth of corynebacteria on the skin surface.

## Results

### *Corynebacterium amycolatum* induces RNase 7 expression in keratinocytes and in an organotypic 3D skin equivalent

**Figure 1:**
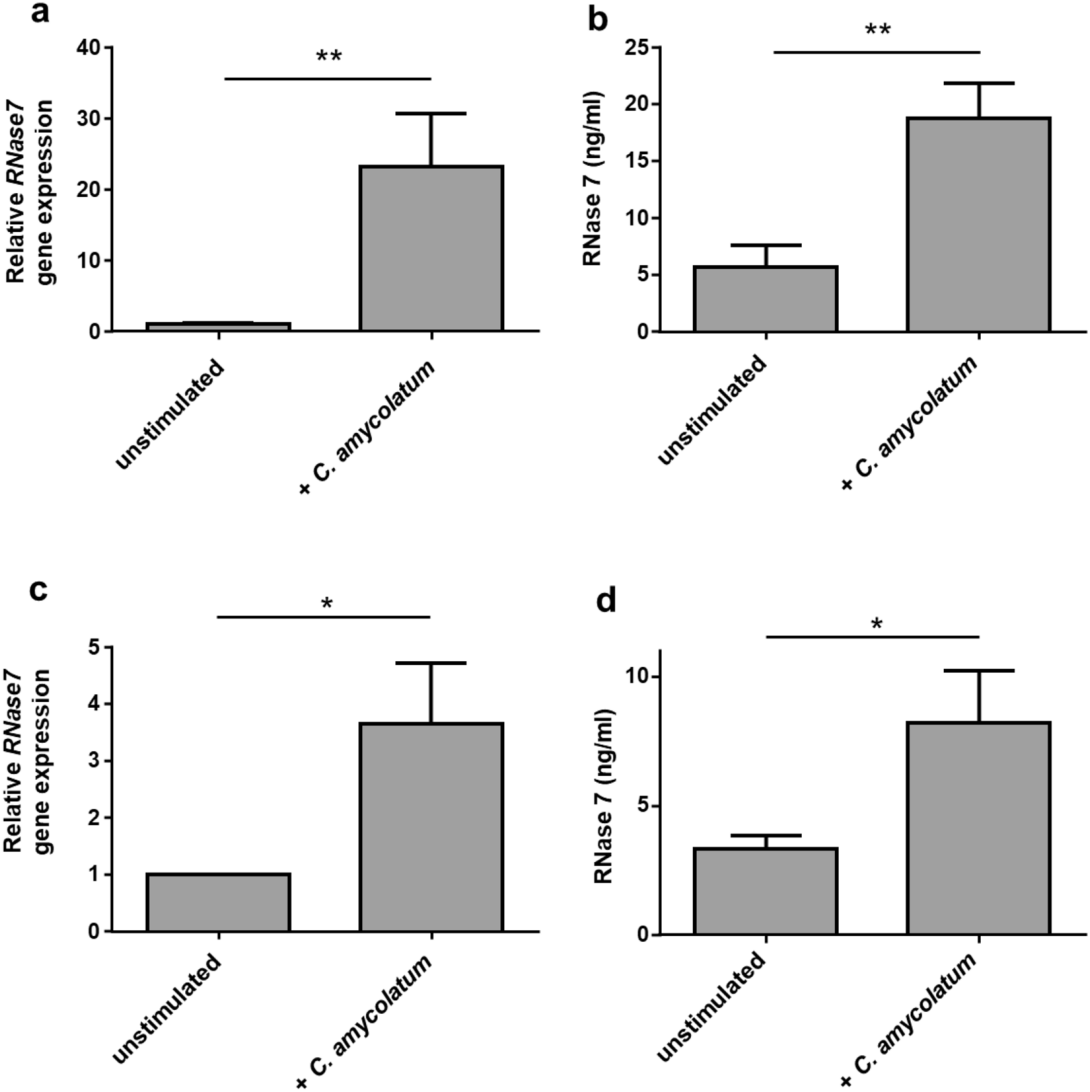
*C. amycolatum* induces RNase 7 expression in primary keratinocytes and in an organotypic skin equivalent. (**a**) Treatment of human primary keratinocytes for 22–24 h with living *C. amycolatum* induced *RNase7* gene expression as measured by real-time PCR. (**b**) ELISA analyses revealed also an increased secretion of RNase 7 by keratinocytes upon stimulation with living *C. amycolatum*. Shown are means ± s.e.m. of six stimulations (**p < 0.01, Student’s t test). (**c**,**d**) An organotypic skin equivalent was treated with living *C. amycolatum* for 22–24 h. (**c**) *RNase7* gene expression was analyzed by real-time PCR and (**d**) RNase 7 secretion was determined by a RNase 7-specific ELISA of the culture medium. Shown are means ± s.e.m. of four different skin equivalents (*p < 0.05, Student’s t test).

**Figure 2:**
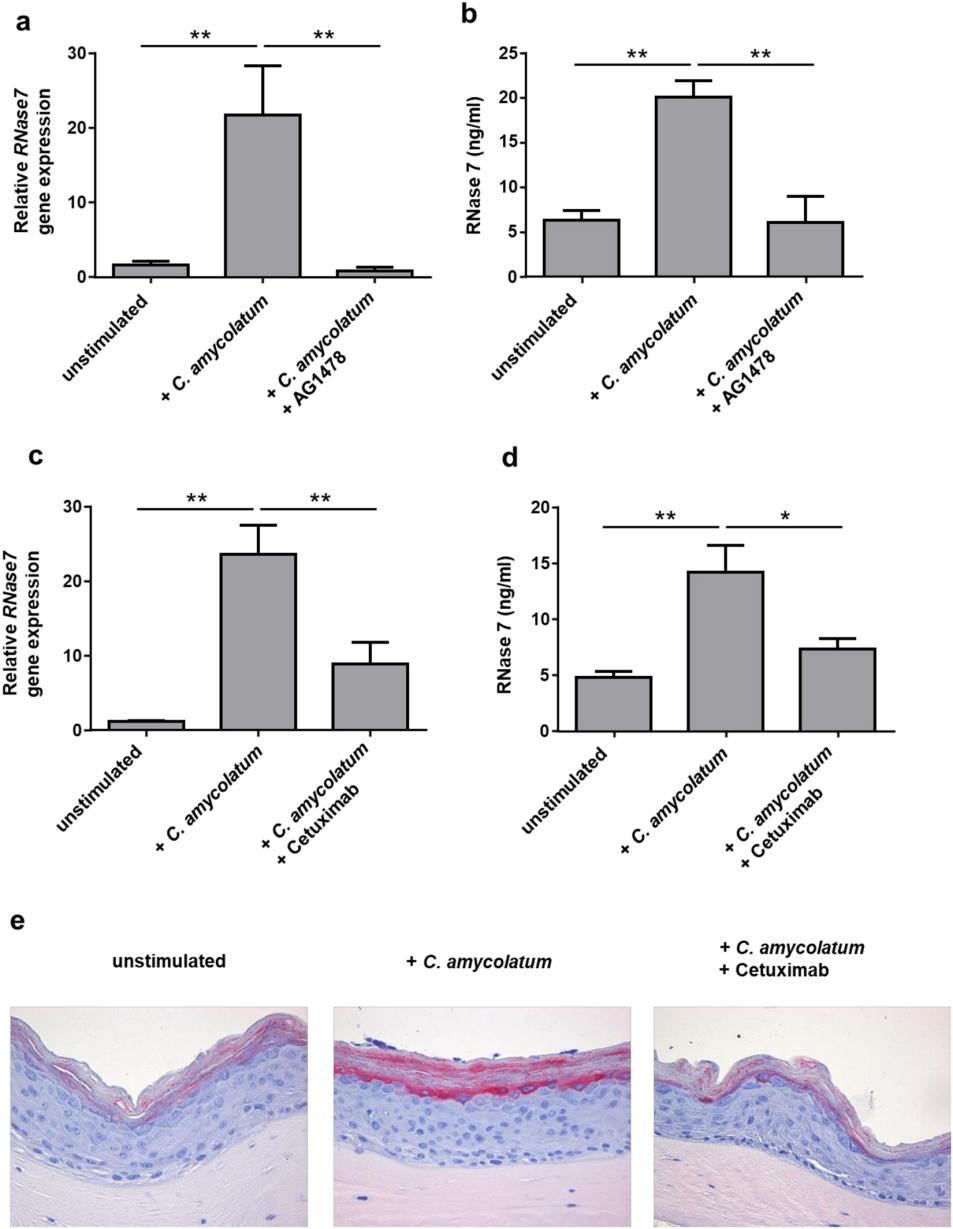
*C. amycolatum*-induced RNase 7 expression in keratinocytes is mediated by the epidermal growth factor receptor (EGFR). Human primary keratinocytes were treated for 24 h with living *C. amycolatum* with or without the selective EGFR inhibitor AG-1478 (10 µM). (**a**) Relative *RNase7* gene expression was analyzed by real-time PCR and (**b**) RNase 7 protein secretion was measured by analysis of the supernatant using an RNase 7 specific ELISA. In a second experimental setup stimulation of the keratinocytes with living *C. amycolatum* was done for 24 h in the absence or presence of the EGFR blocking antibody cetuximab (20 µg/ml). (**c**) Relative *RNase7* gene expression was analyzed by real-time PCR and (**d**) RNase 7 protein secretion was measured by analysis of the supernatant using an RNase 7-specific ELISA. Shown are means ± s.e.m. of nine separate stimulations (*p < 0.05; **p < 0.01, one-way analysis of variance ANOVA using Tukey’s multiple comparison test). (**e**) Analysis of RNase 7 expression by immunostaining in a 3D skin equivalent. The 3D skin equivalent was left unstimulated or stimulated with living *C. amycolatum* for 24 h in the presence or absence of cetuximab (20 µg/ml). Bars represent 50 µM.

Stimulation of human primary keratinocytes with living *C. amycolatum* induced *RNase7* gene expression (Fig. 1a) as well as RNase 7 protein release (Fig. 1b). To analyze whether *C. amycolatum* induces RNase 7 also in a stratified epidermis we used a differentiated organotypic 3D skin equivalent. Application of living *C. amycolatum* for 24 h on the 3D skin equivalent induced *RNase7* gene expression (Fig. 1c) as well as RNase 7 protein release (Fig. 1d). Immunohistochemistry analysis of the 3D skin equivalent revealed also an increased RNase 7 protein expression in the uppermost epidermal layers upon stimulation with *C. amycolatum* (Fig. 2e).

### Induction of RNase 7 by *Corynebacterium amycolatum* in keratinocytes requires functional EGFR

Several reports indicate that the EGFR is critically involved in the induction of RNase 7 by microorganisms^13,14,16^. Therefore we sought to determine whether the induction of RNase 7 by *C. amycolatum* was also dependent on the EGFR. To this end we incubated the keratinocytes with the selective EGFR inhibitor AG-1478. As shown in Fig. 2a,b AG-1478 diminished the induction of *RNase7* gene expression and protein secretion in keratinocytes treated with *C. amycolatum*. In line with these experiments the use of EGFR-blocking antibody cetuximab also significantly decreased the *C. amycolatum*-mediated *RNase7* gene and protein expression in primary keratinocytes (Fig. 2c,d). Immunohistochemistry analysis of a 3D skin equivalent stimulated with living *C. amycolatum* revealed that the *C. amycolatum*-mediated RNase 7 induction was inhibited by treatment with cetuximab (Fig. 2e).

### RNase 7 exhibits antimicrobial activity against *C. amycolatum* and *C. xerosis*

**Figure 3:**
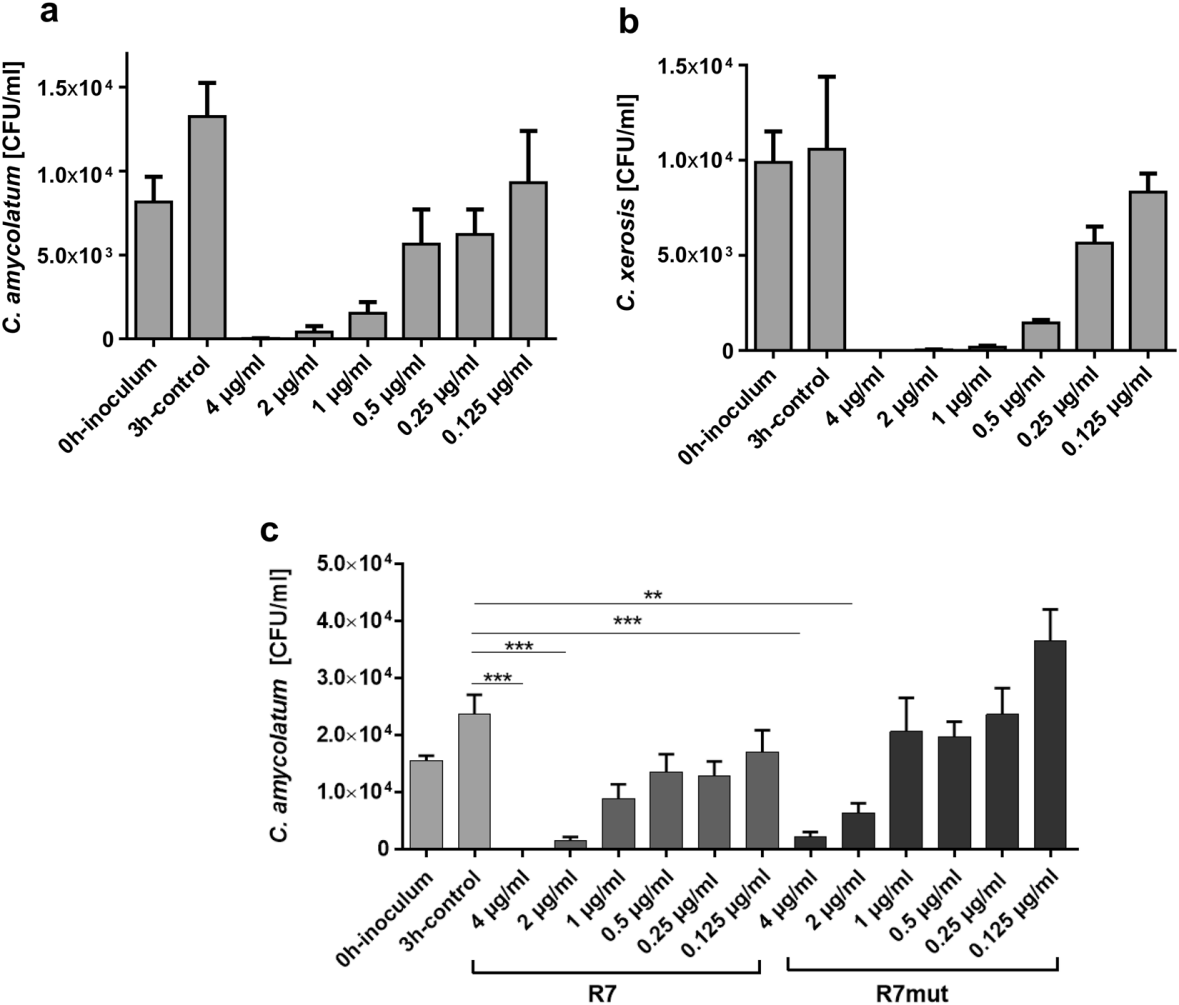
RNase 7 exhibits antimicrobial activity against *C. amycolatum* and *C. xerosis*. (**a**) *C*. *amycolatum* and (**b**) *C*. *xerosis* were incubated with the indicated concentrations of RNase 7 in 10 mM sodium phosphate buffer (pH 7.4) containing 1% BHI and 0.05% BSA. After 3 h incubation time samples were serial diluted and plated on BHI agar plates. The colony forming units (CFU) were counted after overnight incubation at 37 °C. (**c**) The antimicrobial activity of wildtype recombinant RNase 7 (R7) and ribonuclease-deficient recombinant RNase 7 (R7mut) were tested at the indicated concentrations against *C. amycolatum*. Data are means of three independent experiments (**p < 0.01; ***p < 0.001, one-way analysis of variance ANOVA using Tukey’s multiple comparison test).

To determine whether RNase 7 is able to restrict the growth of corynebacteria we incubated *C. amycolatum* and *C. xerosis* with different concentrations of RNase 7 in a microdilution assay for 3 h. As shown in Fig. 3a,b RNase 7 dose-dependently inhibited the growth of *C. amycolatum* and *C. xerosis*. Concentrations lower than 1 µg/ml RNase 7 still inhibited the growth of the bacteria. To investigate whether the enzymatic activity of RNase 7 is necessary for the observed killing activity we used a mutated recombinant RNase 7 without ribonuclease activity. This ribonuclease-inactive RNase 7 variant showed similar activity against *C. amycolatum* as compared to recombinant wild-type RNase 7 (Fig. 3c) indicating that the ribonuclease activity of RNase 7 is not crucial for its antibacterial activity against *C. amycolatum*.

### RNase 7 contributes to the antimicrobial activity of human stratum corneum against *C. amycolatum*

**Figure 4:**
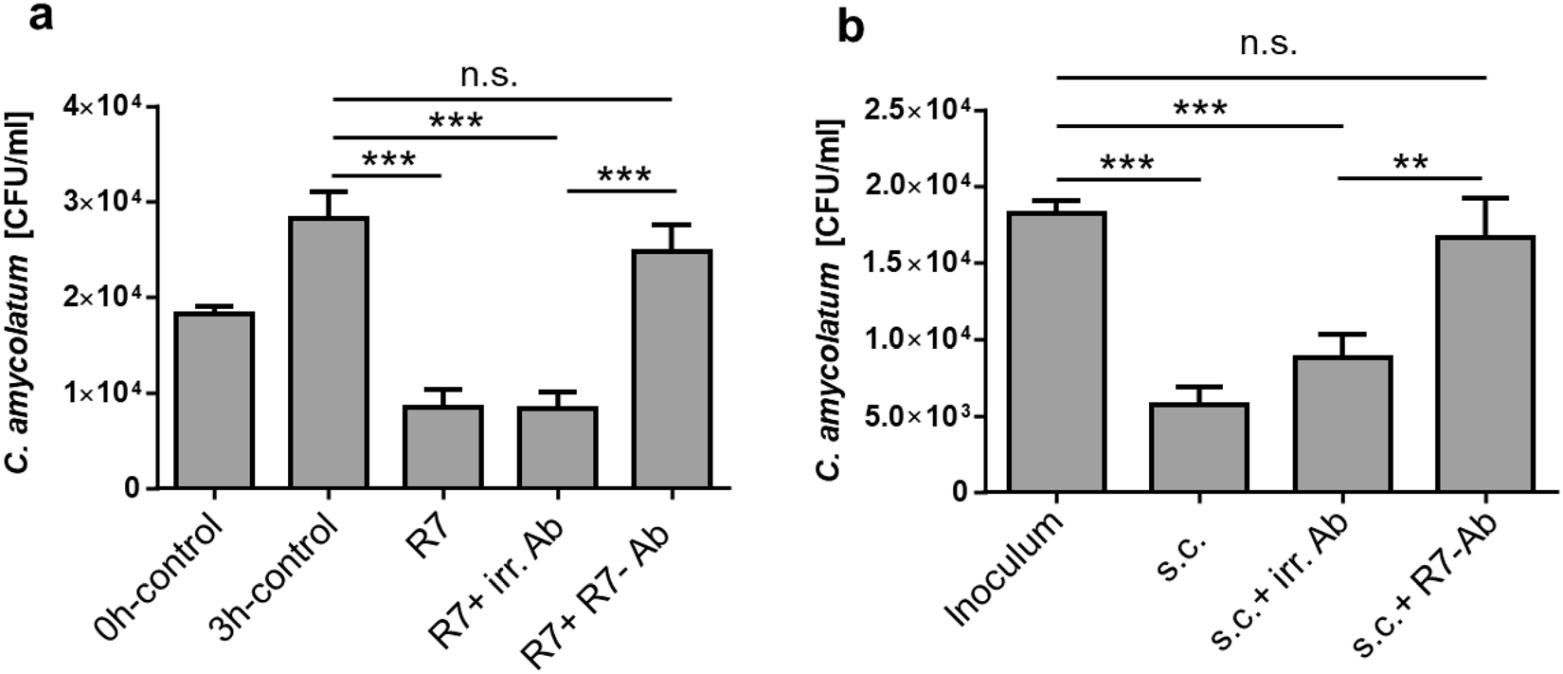
RNase 7 contributes to the antimicrobial activity of human stratum corneum against *C. amycolatum*. (**a**) To test the neutralizing activity of the RNase 7 antibody *C. amycolatum* was incubated for 3 h at 37 °C with RNase 7 (1 µg/ml; R7) together with a specific RNase 7 neutralizing antibody (R7 + R7-Ab). RNase 7 alone (R7) or together with an irrelevant antibody (R7 + irr. Ab) served as positive controls. (**b**) *C. amycolatum* was incubated for 3 h with stratum corneum extract alone (s.c.), in the presence of an irrelevant control antibody (s.c. + irr. Ab) or a specific RNase 7 antibody (s.c. + R7-Ab). After incubation serial dilutions were plated on agar-plates and the colony forming units (CFU) were determined the following day. Shown are means ± s.e.m. of five independent experiments (n.s. = not significant; **p < 0.01; ***p < 0.001, one-way analysis of variance ANOVA using Tukey’s multiple comparison test).

To analyze the functional importance of RNase 7 in cutaneous defense against corynebacteria we used an RNase 7 blocking antibody which neutralized the antimicrobial activity of RNase 7 against *C. amycolatum* whereas an irrelevant control antibody had no influence (Fig. 4a). We then incubated a stratum corneum extract with *C. amycolatum* in the presence of the RNase 7 blocking antibody or in the presence of the irrelevant antibody. The stratum corneum extract was able to control the growth of *C. amycolatum* also in the presence of the irrelevant control antibody. In contrast, inactivation of RNase 7 in the stratum corneum extract by incubation with the RNase 7 blocking antibody led to an outgrowth of *C. amycolatum* (Fig. 4b). These data show that RNase 7 contributes to the capacity of human stratum corneum to control the growth of *C. amycolatum*.

## Discussion

RNase 7 is an antimicrobial protein which is abundantly expressed in keratinocytes and characterized by a broad spectrum of antimicrobial activity^12–14^. This suggests that RNase 7 may play a major role in cutaneous defense. This is in concordance with recent functional studies reporting that RNase 7 contributes to the capacity of the human skin surface to inhibit the growth of *S. aureus, E. faecium* and *P. aeruginosa* ^13,14,17^. In line with these observations it has been reported that high levels of RNase 7 may confer protection against *S. aureus* infection of the skin^18^. Since there is increasing evidence that AMP may also play an important role to control and shape the commensal microbiota we sought to determine whether RNase 7 may help to control cutaneous growth of corynebacteria, inhabitants of healthy normal skin. Our data show that RNase 7 exhibits potent antimicrobial activity against *C. amycolatum* and *C. xerosis*. The use of a recombinant ribonuclease-deficient RNase 7 mutant revealed that the enzymatic activity of RNase 7 was dispensable for its antimicrobial activity against *C. amycolatum*. This is in line with our previous study documenting that the activity of RNase 7 against the gram-positive bacterium *Enterococcus faecium* required no ribonuclease activity^13^.

Our data revealed that concentrations lower than 1 µg/ml RNase 7 already restricted the growth of the corynebacteria suggesting that RNase 7 may play an important role to control the growth of corynebacteria on the skin surface. In support of this hypothesis our results show that the capacity of stratum corneum to inhibit the growth of *C. amycolatum* was reduced when RNase 7 was inactivated by a blocking antibody.

This study demonstrates for the first time that keratinocytes are able to sense the presence of corynebacteria leading to an increased expression of RNase 7. The induction of RNase 7 by *C. amycolatum* in keratinocytes required the EGFR. This is in line with other reports demonstrating a crucial role of the EGFR for the microbial induced expression of RNase 7 in keratinocytes^14,19–21^. It remains to be investigated if the bacteria directly activate the EGFR-pathway as it has been reported for the S*. aureus* protein A-mediated activation of the EGFR^22^ or if the activation of the EGFR follows sensing by a pattern recognition receptor. In this regard a recent report demonstrated the upregulation of Toll-like receptor expression in corneal epithelial cells infected with *Corynebacterium pseudodiphtheriticum* ^23^. This suggests that Toll-like receptors may be involved in the detection of corynebacteria in epithelial cells, a hypothesis which remains to be addressed in further studies.

As mentioned above corynebacteria are abundant commensals on the skin surface. Another abundant skin commensal is *Staphylococcus epidermidis*. Wanke *et al*. reported that *S. epidermidis* induced the expression of RNase 7 in keratinocytes and this induction required functional EGFR^21^. One may speculate that a permanent induction of RNase 7 by skin commensals such as coagulase-negative staphylococci and corynebacteria may increase cutaneous defense by providing constant antimicrobial activity on the skin surface. A failure to adequately induce RNase 7 may contribute to the increased cutaneous infection risk of cancer patients receiving anti-EGFR therapy^24^.

Taken together, our data indicate a novel role of RNase 7 to control the growth of corynebacteria on human skin. It remains to be shown whether a failure to adequately control the growth of *C. amycolatum* and other cutaneous corynebacteria may be associated with a higher risk for infections caused by these bacteria. In addition, it is an intriguing hypothesis that variations in the expression of corynebacteria-controlling AMP such as RNase 7 may influence body odor formation.

## Methods

### Cell culture and stimulation

Primary normal human keratinocytes (NHEK, Promocell, Heidelberg, Germany) were cultured in “Keratinocyte Growth Medium 2” (KGM2) and seeded in 12-well plates for stimulation (passage 3–5). Cells used for stimulation were always 100% confluent. *Corynebacterium amycolatum* and *Corynebacterium xerosis* (clinical isolates from the Institute of Infection Medicine, Kiel; identity verified by MALDI-TOF mass spectrometry (MALDI Biotyper, Bruker, Billerica, MA)) were cultured in brain-heart infusion (BHI) medium (Sigma-Aldrich, St. Louis, MO). For stimulation an overnight culture of bacteria grown at 37 °C under agitation was pelleted by centrifugation (2000 × g, 5 min), washed with phosphate buffered saline (PBS, Biowest SAS, Nuaillé, France) and resuspended and diluted in KGM2 to an optical density (OD_600_) of 0.2 and further diluted 1:10 in KGM2 cell culture medium for stimulation. Stimulation (500 µl each well) was carried out for 20–22 h or for the indicated time period. To analyze the impact of the EGFR a specific monoclonal anti-EGFR antibody cetuximab (20 µg/ml, Merck, Darmstadt, Germany) as well as the specific EGFR inhibitor AG-1478 (10 µM, Tyrphostin, ENZO LifeScience, Lörrach, Germany) were used.

### Organotypic 3D skin equivalent

The organotypic 3D skin equivalent was generated as previously described^14^. The 3D skin equivalent was stimulated with 20 µl *C. amycolatum* diluted in KGM2 (OD_600_ of 0.2) as described above. In order to block the EGFR 20 µg/ml cetuximab was added to the culture medium in the external wells 45 min before stimulation. After stimulation for 24 h two biopsies were taken from each 3D skin equivalent using a 6 mm biopsy punch. One biopsy was embedded in paraffin for immunohistochemical analysis and the other biopsy was used for RNA isolation. The KGM2 medium in the external wells was harvested for ELISA.

### RNA isolation and cDNA synthesis

Total RNA of the keratinocytes and the 3D skin equivalent was isolated using 500 µl of the RNA isolation reagent Crystal RNAmagic according to the manufacturer’s protocol (BiolabProducts, Gödenstorf, Germany). The isolated RNA was dissolved in H_2_O and 0.5 µg total RNA was reversed transcribed to cDNA using an oligo (dT)18 primer and 50 Units Maxima Reverse Transcriptase (Thermo Fisher Scientific, Waltham, MA) according to the supplier’s protocol.

### Real-time PCR analysis

Quantitative real-time PCR was done in a StepOnePlus Real Time PCR System (Applied Biosystem, Carlsbad, CA) as previously described^25^ using SYBR Premix Ex Taq II (TaKaRa Bio, Saint-Germain-en-Laye, France) and cDNA corresponding to 10 ng total RNA as template. The following intron-spanning primers were used: RNase 7: 5′-GGA GTC ACA GCA CGA AGA CCA-3′ (forward primer) and 5′-CAT GGC TGA GTT GCA TGC TTG A-3′ (reverse primer) and the house keeping gene RPL38 (ribosomal protein L38): 5′-TCA AGG ACT TCC TGC TCA CA-3′ (forward primer) and 5′-AAA GGT ATC TGC TGC ATC GAA-3′ (reverse primer). Standard curves were generated for each primer pair using serial dilutions of template cDNA. Relative *RNase7* gene expression is given as a ratio between expression of *RNase7* and *RPL38* gene expression.

### ELISA

Secreted RNase 7 protein levels in the cell culture supernatants were measured by a specific RNase 7 ELISA as previously described^13^. The detection range of the RNase 7 ELISA was between 0.3 ng/ml and 40.0 ng/ml.

### Immunostaining

The organotypic 3D skin equivalent was embedded in paraffin and immunostaining was performed as described^26^. Briefly, a self-generated goat anti-RNase 7 antibody^13^ was used followed by biotinylated rabbit anti-goat IgG antibody (DakoCytomation, Glostrup, Denmark) and avidin/biotinylated enzyme complex (Vectastain ABC-AP staining-kit, Vector laboratories, Peterborough, UK) and a red alkaline phosphatase substrate (Red AP; Vector Red, Vector laboratorie, Burlingame, Ca). Slides were counter stained with hematoxylin and mounted with Eukitt (Poly(butyl methacrylate-co-methyl methacrylate); O. Kindler, Freiburg, Germany). The use of pre-immune serum instead of an antibody served as negative control.

### Antimicrobial Assay

Corynebacteria were grown in BHI medium overnight at 37 °C until reaching an OD_600_ of 0.2. This culture was diluted 1:1000 in 10 mM sodium phosphate buffer containing 2% BHI medium and 0.1% BSA (bovine serum albumin, Sigma-Aldrich). 25 µl of this bacteria solution was mixed with 25 µl 10 mM sodium phosphate buffer containing different concentrations of recombinant RNase 7 or stratum corneum extract prepared as previously described^13^. The use of human stratum corneum derived from the heel was approved by the Ethics Committee at the Medical Faculty of the Christian-Albrechts-University, Kiel, Germany (A104/06) in concordance with the Declaration of Helsinki protocols and donors have given informed consent. Incubation was carried out at 37 °C for 3 h followed by plating serial dilutions on BHI agar plates to analyze colony forming units (CFU) after overnight incubation at 37 °C. In some experiments incubation was performed in the presence of a specific RNase 7 blocking antibody (0.5–0.1 mg/ml) or an irrelevant antibody as described^13,14^.

To investigate whether the ribonuclease activity of RNase 7 is responsible for its antibacterial activity against *C. amycolatum* we used a recombinant mutant RNase 7 without ribonuclease activity as previously described^13^.

### Data Availability

The datasets generated during the current study are available from the corresponding author on reasonable request.
